# Combination of HPLC-Q-TOF-MS/MS, Network Pharmacology, and Molecular Docking to Reveal the Mechanism of Apple Pollen in the Treatment of Type 2 Diabetes Mellitus

**DOI:** 10.1155/2022/3221196

**Published:** 2022-05-24

**Authors:** Yiwen Wang, Ping Chen

**Affiliations:** ^1^Department of Pharmacy, Shaanxi University of Chinese Medicine, Xianyang, Shaanxi 712046, China; ^2^Shaanxi Academy of Traditional Chinese Medicine, Xi'an, Shaanxi 710003, China

## Abstract

Studies have found that apple pollen can restrain the activity of amylase. Therefore, we speculate that it may be prescribed to treat patients with type 2 diabetes mellitus (T2DM), while its chemical and pharmacologic profiles remain to be further explained. In this study, the potential bioactive compounds of apple pollen and the underlying mechanism of action were investigated by performing chemical and network pharmacology analysis. Therefore, HPLC-QTOF-MS/MS analysis based on chemical compound libraries was applied in identifying the chemical profiles of apple pollen and network pharmacology was adopted for predicting the potential targets of the active components of apple pollen. Initially, the chemical map of apple pollen was identified and characterized. Secondly, the potential targets of active compounds of apple pollen were predicted with the Swiss Target Prediction and PharmMapper databases, whereas targets of T2DM were collected from the GeneCards and OMIM database. Thereafter, the target of active compounds and T2DM targets established common targets using Venn. Afterwards, the common targets were imported into the STRING database in order to construct the protein-protein interaction (PPI) network and select the core targets of apple pollen treatment of T2DM. In addition, GO and KEGG signaling pathway enrichment analyses were conducted on the selected core targets using the DAVID database. As a result, totally 28 compounds were identified. Meanwhile, network pharmacological analysis showed that 3-hydroxy-3-methyl glutaric acid, 5-hydroxyindoleacetic acid, DL-3-phenyllactic acid, isorhamnetin-3-glucoside-4′-glucoside, isorhamnetin-3-O-glucoside, syringetin-3-O-galactoside, rhamnetin, m-coumaric acid, quercitrin, isorhamnetin-3-galactoside-6″-rhamnoside, and kaempferol-3-O-alpha-L-arabinoside might be the active compounds of apple pollen. Moreover, AKT1, PPARG, SRC, EGFR, CASP3, ESR1, and the other potential core targets might be involved in the treatment of T2DM by modulating the following pathways, containing insulin resistance, hepatitis C, pancreatic cancer, insulin signaling pathway, TNF signaling pathway, and PI3K-AKT signaling pathway. Quercitrin, kaempferol, and isorhamnetin-3-O-glucoside bound most stably to AKT1. Isorhamnetin-3-O-glucoside and quercitrin bound most stably to SRC. In addition, arachidonic acid bound most stably to PPARG.

## 1. Introduction

Type 2 diabetes mellitus (T2DM) is the main subtype of diabetes mellitus (DM), with insulin resistance (IR) being the main pathological feature. T2DM is characterized by the disorders of glucose metabolism, glucose metabolism-related enzymes, and lipid metabolism. The main complications include cardiovascular disease (CVD), renal failure (RF), peripheral neuropathy, and obesity, causing serious health damage and economic burdens on the patients [1, 2]. At present, insulin and its analogues such as sulfonylureas and glinides are the main drugs used to treat T2DM [3]. However, these drugs will cause a variety of side effects such as cardiotoxicity and psychotoxicity. Therefore, it is of necessity to explore the safe and effective anti-T2DM drugs for the clinical application.

Pollen is a kind of natural advanced nutrition worthy of development and research, not only rich in amino acids, vitamins, nucleic acids, flavonoids, unsaturated fatty acids, protein active enzymes [4], etc., but also in a reasonable ratio [5]. Due to its good cosmetic effect, it is called “edible cosmetics” by women [6–10]. In addition, it has an extremely important role in preventing human aging, promoting metabolism, and regulating human functions [11]. Apple pollen (*Malus pumila* Mill.) is a kind of apple pollen powder, which is extensively distributed throughout China and is usually collected from April to May in spring. Apple pollen has been reported to possess extensive pharmacological activities [12–15], such as preventing myocardial infarction (MI), strengthening the immune system, preventing aging, treating CVDs, inhibiting tumor, and protecting the liver [6]. Some scholars [10] performed comparative tests on the effects of various pollen on enhancing immune function, including body weight, thymus and spleen weight, lymphocyte transformation, macrophage phagocytosis test, NK cell activity, peripheral blood IgG, spleen plaque hemolysis test, lactate dehydrogenase, and arginase of peritoneal macrophages. Clinical evidence demonstrates that pollen is effective in improving human immune function. Consequently, it has a favorable clinical application and development prospect [16]. Additionally, studies have also shown that pollen can lower intestinal absorption of cholesterol, increase excretion, inhibit the proliferation of high-fat aortic smooth muscle cells, show its SMC proliferation, and exert an important role in reducing plaque formation, as well as regulating the metabolism of prostacyclin and thromboxane AZ. It also shows that it can regulate the transformation of prostaglandin metabolic enzymes as well as the absorption and excretion of cholesterol. Pollen can inhibit platelet aggregation, improve blood fluidity, activate the function of monocyte macrophages, and protect cell intima, also preventing fibrin damage and other systemic pharmacological effects. Clinically, it generates a good effect on cardiovascular diseases [9]. The main chemical components of apple pollen [17, 18] include flavonoids, organic acids, polysaccharides, amino acids, vitamins, and mineral elements. In the present study, two major varieties of apple pollen (Qinguan and Gala pollen) were selected as the objects due to their extensive source, high yield, and good taste.

**Figure 1:**
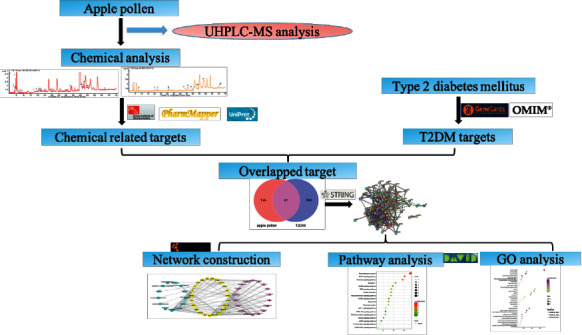
Flow chart of network pharmacology research scheme of apple pollen in the treatment of T2DM.

High performance liquid chromatography coupled with electrospray ionization and quadrupole time-of-flight-mass spectrometry (HPLC-Q-TOF-MS/MS) is a powerful and reliable analytical technique developed to identify natural products [19], which is ascribed to its advantages of efficient separation ability of HPLC and highly sensitive detection of MS [20]. Till the present, HPLC-Q-TOF-MS/MS has never been reported for the identification and comparison of chemical compositions in Qinguan and Gala pollen. Therefore, this study mainly aimed to develop a rapid and effective method for the identification and comparison of major components in apple pollen based on the HPLC-QTOF-MS/MS technique. Then, the quality markers of apple pollen for treating T2DM are further selected from the detected components by network pharmacology. Based on the obtained findings, active constituent and the mechanism of action of apple pollen for treating T2DM will be revealed. The design of this experiment is displayed in Figure 1.

## 2. Materials and Methods

### 2.1. Materials

The Agilent 1200 HPLC system interfaced with Agilent 6520 hybrid quadrupole time of flight mass spectrometer (Agilent Technologies, USA) was utilized in this study. The system was operated under the control of MassHunter Acquisition software (version B.04.00).

Both methanol and formic acid (LC-MS grade) were purchased from Fisher Scientific (Leicestershire, UK), purified water was provided by Hangzhou Wahaha Group (Hangzhou, China), and ethanol (AR grade) was obtained from Tianjin Tianli Chemical Reagents Ltd. (Tianjin, China).

Qinguan pollen (batch number: 20140410) and Gala pollen (batch number: 20140401) were obtained from apple demonstration areas in the Northwest Agriculture and Forestry University (Baishui, Shaanxi Province; Lingbao, Henan Province).

### 2.2. HPLC-QTOF-MS/MS Analyses

#### 2.2.1. HPLC and MS Conditions

Chromatographic separation was performed using a Boston Green ODS-C18 column (4.6 × 250 mm, 5 *μ*m). The mobile phase consisted of A (0.1% formic acid in water) and B (0.1% formic acid in methanol). The following gradient program was adopted for separation: 0∼15 min, 10%∼20%; 15∼45 min, 20%∼40% B; 45∼75 min, 40%∼80% B; 75∼80 min, 80%∼90% B; and 80∼85 min, 90%∼100% B. The injection volume was 10 *μ*L, the column temperature was set at 25°C, and the solvent flow rate was set at 0.8 mL·min^−1^.

Mass spectrometer was operated in the negative electrospray ionization mode, and spectra were recorded by scanning the mass range from m/z 50 to 1100. The flow rate of drying gas (N2) was set at 8 L/min. Meanwhile, the heated capillary temperature was set at 350°C and the nebulizer pressure was set at 35 psi. Besides, the source parameter capillary voltage was set at 3500 V, the fragmenting voltage was 135 V, and the split ratio was 1 : 3. The collision energy for MS/MS analysis was tested within the range of 20–40 eV [21].

#### 2.2.2. Sample Preparation

Approximately 1.0 g of each sample (Qinguan pollen and Gala pollen) was accurately weighed and put into a capped 100 mL conical flask. Thereafter, each respective sample was extracted with 50 mL ethanol by reflux extraction for 1 h. Then, the extract was filtered and dried, while the residue was reconstituted with 50 mL methanol and filtered through a 0.25 *μ*m membrane filter before the injection into the HPLC system. The sample injection volume was 10 *µ*L.

#### 2.2.3. Identification of Compounds

The accurate mass data of molecular ions were processed by adopting the Mass Hunter Acquisition software (version B.04.00). The obtained parent ion information and a large number of daughter ion fragments were analyzed by MassHunter software. The molecular formula and molecular weight were deduced by the “molecular search function” and matched with the MassBank standard spectrum to deduce the chemical structure and determine the final structure of the compound. The total ion chromatograms of Qinguan and Gala apple pollen obtained are illustrated in Figure 1.

### 2.3. Network Pharmacology

#### 2.3.1. Target Prediction

Initially, the canonical SMILES information of each identified compounds was obtained by the NCBI PubChem database (https://www.ncbi.nlm.nih.gov/pubmed/) and then uploaded into the SwissTargetPrediction database (https://www.swisstargetprediction.ch/) [9] and PharmMapper (https://www.lilab-ecust.cn/pharmmapper/). Obviously, the species was limited to “*Homo sapiens*” for target prediction and the minimum interaction threshold was set to “highest possible” >0.6 by comparing with the known active compounds. Secondly, T2DM-related targets were obtained from the DrugBank database (https://go.drugbank.com) and GeneCards database (https://www.genecards.org/) based on the key word search of “Type 2 Diabetes Mellitus.” Besides, the duplicate targets were eliminated.

#### 2.3.2. Construction of the Target Protein-Protein Interaction (PPI) Network

The PPI network was constructed based on the STRING database (https://string-db.org/) by incorporating the overlapping targets between the candidate compound targets and the T2DM-related targets.

#### 2.3.3. Gene Ontology (GO) Functional Annotation and Kyoto Encyclopedia of Genes and Genomes (KEGG) Pathway Enrichment Analyses

GO functional annotation and KEGG pathway enrichment analyses of the common target genes were conducted using the DAVID database platform (https://david.ncifcrf.gov) [22, 23]. The results were presented with online analysis as a bar chart and bubble chart, respectively.

#### 2.3.4. Molecular Docking

The core component target and ligand files were retrieved from the chemical database (Pub Chem) and the protein database (RCSB-Pdb). The Autodock Vina software was adopted for molecular docking of the core component target.

#### 2.3.5. Construction of the Active Component-Common Target Gene Pathway Network

The pharmacological network of “chemicals-targets-pathways” was constructed to screen the potential chemical components related to the therapeutic effects on T2DM (Figure 1). The active component-disease-target gene pathway interaction network was constructed by the Cytoscape 3.7.1 software.

## 3. Results and Discussion

### 3.1. Chemical Profiles of Apple Pollen Detected by UHPLC-Q/TQF-MS

**Figure 2:**
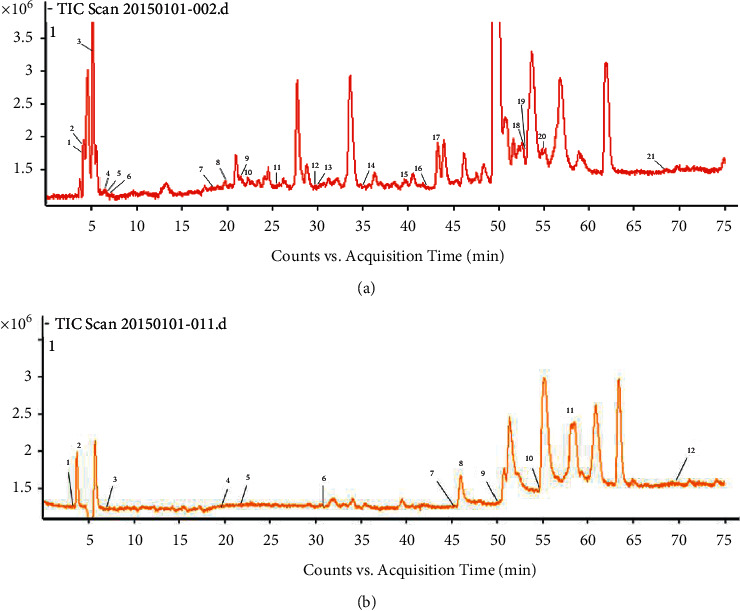
HPLC-QTOF-MS total ion chromatograms of apple pollen. (a) Qinguan pollen. (b) Gala pollen.

The monitoring ion for detection and analysis is shown in Figure 2. In the current work, altogether 32 components from Qinguan pollen and Gala pollen were characterized by UHPLC-Q/TOF-MS analysis (Tables 1 and 2).

A total of 28 compounds were identified, which mainly included flavonoids and organic acids (Tables 1 and 2). Among them, 17 compounds were identified and characterized for the first time in apple pollen, including malvidin, glucuronic acid, N,N-dimethyl-N′-phenylsulfamide, amobarbital, 3-hydroxy-3-methyl glutaric acid, 7-methylguanine, 5-hydroxyin doleacetic acid, L-tryptophan, coumarinic acid-*β*-D-glucoside, quercitrin, rhamnetin, m-coumaric acid, DL-3-phenyllacticacid, isorhamnetin-3-glucoside-4′-glucoside, isorhamnetin-3-O-glucoside, syringetin-3-O-galactoside, isorhamnetin-3-galactoside-6″-rhamnoside, and kaempferol-3-O-alpha-L-arabinoside [18, 24, 25].

### 3.2. Absorption Parameters of Components

**Figure 3:**
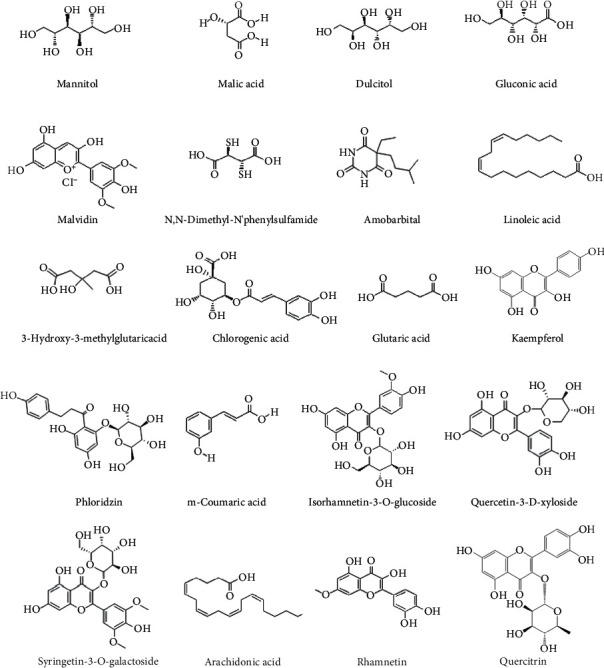
Structures of 21 components.

**Figure 4:**
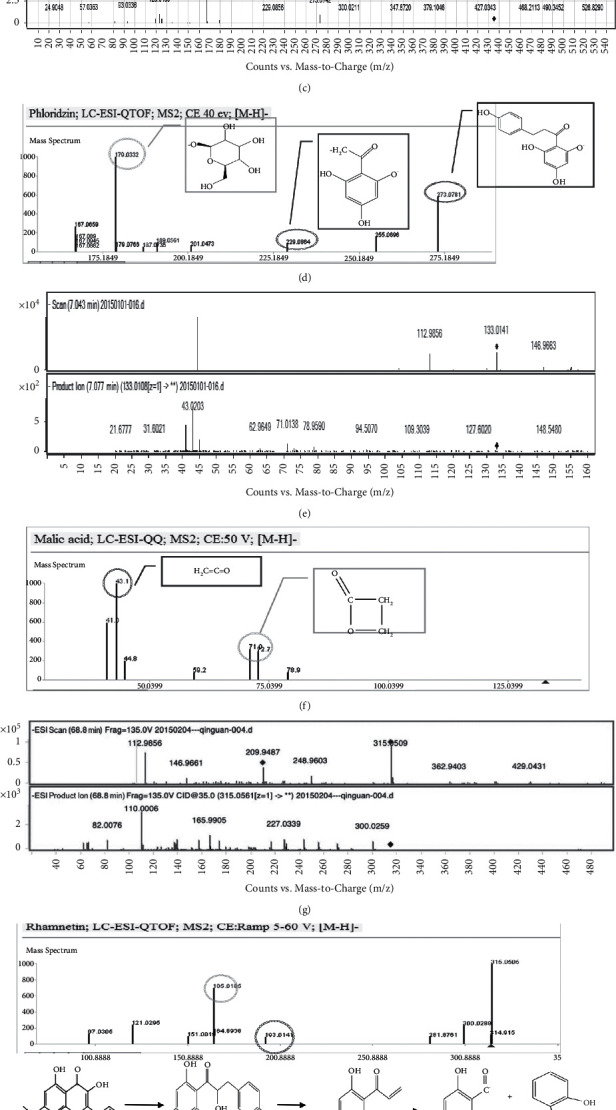
Partial analytic diagram. (a) Chlorogenic acid secondary mass spectrum. (b) Chlorogenic acid mass spectrum. (c) Phlorizin secondary mass spectrum. (d) Phlorizin mass spectrum. (e) Malic acid secondary mass spectrum. (f) Malic acid mass spectrum. (g) Rhamnetin secondary mass spectrum. (h) Rhamnetin mass spectrum. (i) Quercitrin secondary mass spectrum. (j) Quercitrin mass spectrum.

Based on a computer prediction method to calculate the identified compounds of apple pollen, this study obtained absorption parameters that could determine whether the chemical compositions could be absorbed.  Table 3 showed the specific absorption parameters of all of the components [26]. The data indicated that there were a total of 18 chemical compositions (Figures 3 and 4) that satisfied the principles of drug absorption (GI absorption is high and at least two of the five Druglikeness were yes). Although the relative molecular masses of those compounds were greater than 500, we could import these glycosides' aglycones into PharmMapper in order to obtain the relevant parameters. According to the results, both of these components were consistent with the five principles of drug absorption, and thus we considered that these 7 chemical compositions could be absorbed in the body.

### 3.3. Screening of Chemical Ingredient Targets in Apple Pollen for Treating T2DM

**Figure 5:**
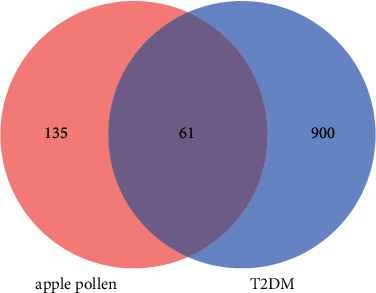
Venn diagram of apple pollen-type 2 diabetes mellitus targets.

By employing Swiss Target Prediction and PharmMapper, a total of 196 targets were predicted. Additionally, totally 961 T2DM target genes were uncovered in the GeneCards database and OMIM database. Later, the above genes were intersected in the Venny, and 196 target genes for Apple pollen for T2DM were obtained (Figure 5). At the same time, 61 targets were also acquired after intersection.

### 3.4. Construction and Analysis of the Target PPI Network

**Figure 6:**
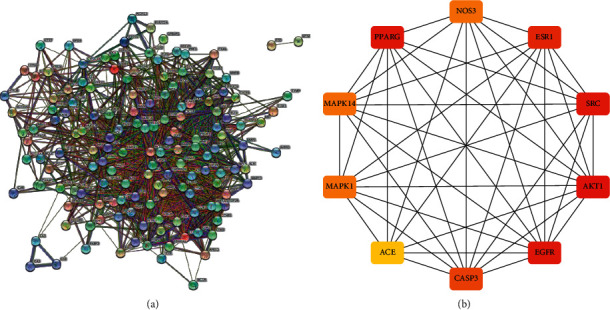
(a) PPI network of the common target genes. The nodes represent target genes; the stuffing of the nodes represents 3D structure of target genes; the edges represent target genes-target genes associations; the colors of the edges represent different interactions; cyan and purple represent known interactions; green, red, and blue purple represent predicted interactions; chartreuse, black, and light blue represent others. (b) 10 hub targets.

After comparatively analyzing 28 component targets and T2DM disease targets, 37 common potential targets were identified for apple pollen. The STRING database is a commonly used tool to predict PPI and produce the integrated and objective association networks. With the common protein targets as the input for network visualization, a diversified PPI network was created, which systematically summarized the interactions of apple pollen targets associated with T2DM treatment (Figure 6(a)). The hub protein was obtained by using CytoHubba plug-in. See Figure 6(b). The 10 hub proteins were AKT1, EGFR, SRC, PPARG, ESR1, CASP3, Mapk14, mapk1, NOS3, and ace from large to small.

### 3.5. Screening of Key Pathways in Apple Pollen for Treating T2DM

Thereafter, the drug-disease intersection genes were introduced into the DAVID database. Then, the target gene name list was input, the species was limited to “*Homo sapiens*,” the target gene name was modified to official gene symbol, and the threshold was set at *P* < 0.01. Thereafter, GO functional annotation and KEGG pathway enrichment analyses were performed.

**Figure 7:**
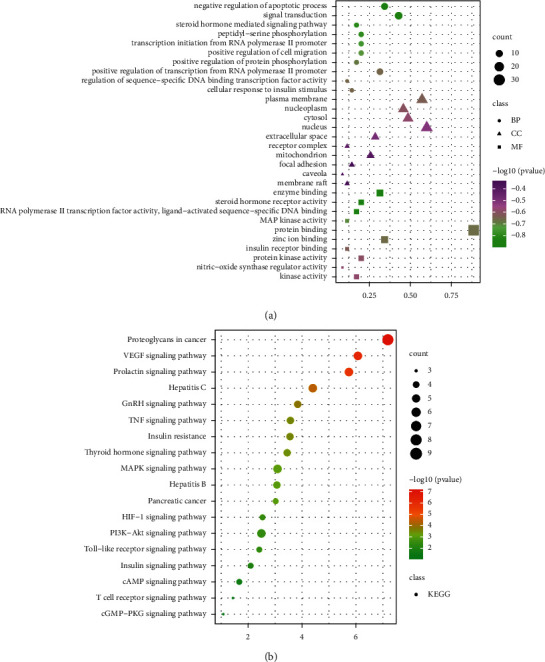
GO function and KEGG signaling pathway analysis of the core targets of apple pollen. (a) GO function analysis of the core targets genes. (b) KEGG enrichment analysis of the core targets apple pollen.

GO analysis of the common targets revealed that the biological process (BP) was mainly related to negative regulation of apoptotic process, signal transduction, steroid hormone mediated signaling pathway, peptidyl-serine phosphorylation, transcription initiation from RNA polymerase II promoter, positive regulation of transcription from RNA polymerase II promoter, and cellular response to insulin stimulus (Figure 7(a)).

KEGG pathway enrichment analysis of the aforementioned common target genes is presented in Figure 7(b).

### 3.6. Active Component-Target-Gene-Pathway Interaction Network

**Figure 8:**
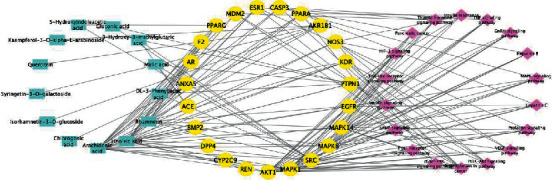
The association network of potential genes of apple pollen. The square represents 14 chemical components, the circle represents 22 genes, and the diamond represents 18 pathways.

The network diagram of “Active Component Target-Gene-Pathway” was constructed in Cytoscape software (Figure 8). According to the analysis results, the network consisted of 54 nodes and 160 edges in total, and each chemical acted on multiple T2DM targets, reflecting the “multicomponent and multitarget” mechanism in the treatment of T2DM. Specifically, the square, circular, and diamond nodes represent targets, apple pollen components, and pathways, respectively, and each node size was proportional to its degree.

### 3.7. Molecular Docking Verification

**Figure 9:**
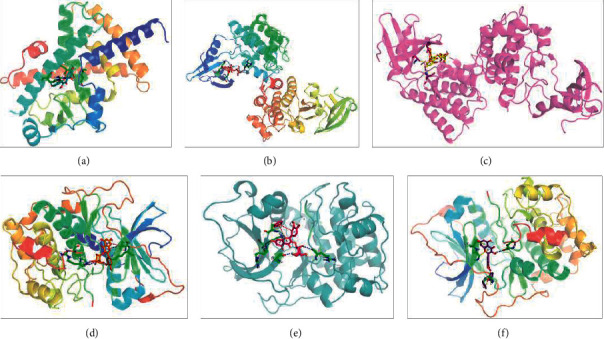
Molecular docking diagram. 3D diagram of arachidonic acid-PPARG (a), quercitrin-SRC (b), and isorhamnetin-3-O-glucoside-SRC (c). 3D diagram of quercitrin-AKT1 (d), isorhamnetin-3-O-glucoside-AKT1 (e), and kaempferol-AKT1 (f).

The core components arachidonic acid, quercitrin, isorhamnetin-3-O-glucoside, and kaempferol were molecularly docked with the core targets AKT1, PPARG, and SRC and when the binding bond energy was <−5 kcal/mol, indicating good docking results for both. The molecular docking model was plotted using PyMOL [27] (Figure 9).

## 4. Discussion

Over the past 30 years, the number of DM cases in China has increased significantly. Among them, T2DM accounts for 97.3%, whereas type 1 diabetes mellitus (T1DM) takes up nearly 5%, and other types account for only 0.7% [4]. T2MD is a chronic metabolic disease, which can also generate a variety of complications, such as diabetic nephropathy, diabetic foot, diabetic encephalopathy, and even physical disability or death. Its etiology is complex and its pathogenesis remains to be further elucidated. At present, insulin resistance, inflammatory cytokines, and DNA methylation are mostly considered to be associated with its etiology. We have experimentally confirmed that the alcoholic and aqueous extracts of apple pollen exert an inhibitory effect on *α*-amylase. Therefore, we predict that apple pollen generates a therapeutic effect on T2DM.

The HPLC-Q-TOF-MS/MS technique [28] helped to identify the chemical compositions in two kinds of apple pollen accurately and rapidly. At first, we employ the separation system and the physical and chemical identification methods. A previous study in apple pollen has tentatively confirmed the main chemical classes, such as flavonoid glycosides and organic acids [29]. The carboxyl and phenolic hydroxyl of organic acid molecules can easily form stable oxygen anion, while mass spectrometry in negative electrospray ionization mode allows obtaining more information than that in the positive mode. With better peak response in negative mode than that in positive mode, flavonoid glycoside compounds have relatively large polarity [30]. Therefore, this study chose negative electrospray ionization mode. We successfully identified a total of 26 compounds in Qinguan pollen and Gala apple pollen using HPLC-Q-TOF-MS/MS technique. According to the obtained results, the selected components of apple pollen showed a high binding activity, which might be used as the potential target genes of apple pollen for treating T2DM. These constituents primarily included arachidonic acid, quercitrin, kaempferol, and isorhamnetin-3-O-glucoside.

In addition to active ingredients, we also successfully predicted drug targets of apple pollen. The major targets included AKT1, PPARC, EGFR, and SRC. AKT1 is one of 3 closely related serine/threonine-protein kinases (AKT1, AKT2, and AKT3) called the AKT kinase, regulating numerous processes including metabolism, proliferation, cell survival, growth, and angiogenesis. AKT is responsible for the regulation of glucose uptake by mediating insulin-induced translocation of the SLC2A4/GLUT4 glucose transporter to the cell surface (by similarity) [30]. PPARG binds nuclear receptor for peroxisome proliferators, such as hypolipidemic drugs and fatty acids. Once activated by a ligand, the nuclear receptor binds to DNA specific PPAR response elements (PPRE) and modulates the transcription of its target genes, such as acyl-CoA oxidase. As a result, it controls the peroxisomal beta-oxidation pathway of fatty acids. PPARG is the key regulator of adipocyte differentiation and glucose homeostasis [31].

Analysis of KEGG enrichment pathways identified totally 17 pathways that are closely related to the development of T2DM. Meanwhile, the relationship between several of these pathways and T2DM and its complications has been verified by experimental or clinical studies. For example, patients with hepatitis B or C are more likely to develop diabetes, and the possible mechanism lies in the fact that hepatitis B virus infection can lead to abnormal liver metabolism. Consequently, insulin resistance and hepatitis B virus can lead to hepatic glucose metabolism by impairing liver function and hepatitis B virus can cause abnormal liver glucose metabolism by impairing liver function [32]. Besides, hepatitis C virus can affect insulin signaling, influence lipid metabolism, and damage islet cells, thus contributing to the development of diabetes [33]; HIF-*α* is closely related to the development of diabetes and its complications [34]. A large amount of HIF-*α* will accumulate in cells under high glucose hypoxia, aggravating the ischemic-hypoxic state of diabetic patients and promoting the development of diabetes. TNF can induce apoptosis by promoting the release of inflammatory factors [35], and TNF-*α* is closely related to insulin resistance [36]. The mechanism may be associated with TNF-*α* inhibiting insulin signaling by activating the NF-*κ*B signaling pathway [37]. In addition, serum TNF-*α* levels are positively correlated with the course of diabetic nephropathy, and the possible mechanism is that TNF-*α* damages diabetic glomerular tissue by producing inflammatory response cytokines and increasing microvascular permeability [29]. The PI3K-AKT [38] signaling pathway is the main pathway associated with insulin signal transduction, which can regulate glucose homeostasis and lipid metabolism by mediating the production of growth factors. Inhibition of this pathway can lead to insulin resistance and subsequently suppress T2DM [39]. Apple pollen can exert a role in treating T2DM by intervening in the above pathways. For example, quercetin can lower blood glucose in T2DM rats by reducing the level of oxidative stress, which can also effectively inhibit TNF in vascular smooth muscle cells-*α* and monocyte chemoattractant protein-1 (MCP-1).

Altogether 28 compounds were successfully identified in Qinguan pollen and Gala apple pollen, including flavonoids and organic acids. Among them, 17 compounds were identified for the first time in apple pollen, while the other 9 were previously reported [25].

From the obtained total ion chromatograms of Qinguan pollen and Gala apple pollen, it was found that a few of chromatogram peaks did not match with the corresponding compounds, indicating the presence of some unknown chemical compositions in apple pollen, which should be further investigated.

## 5. Conclusions

To conclude, different from chemical drugs, TCM is a multi-component and complex drug. Thus, it is challenging to pinpoint the mech-anisms of action of TCM. Each herb may contain many active ingredients that have single or multiple targets. As a result, it is difficult to pinpoint the mechanisms of action of TCM. However, in the traditional research of network pharmacology, the compounds are mostly collected from databases. Besides, some compounds cannot be detected in some TCM, which may yield false positive results.

In this study, we use HPLC-Q-TOF-MS/MS based on multiple in-house chemical libraries coupled with network pharmacology, which was utilized to characterize the chemical compounds of apple pollen and investigate its underlying mechanism in treating T2DM.

A total of 2 compounds were identified in apple pollen, among which, 20 were considered as the potential compounds. Apart from that, a total of 510 targets were predicted, and AKT1, PPARG, EGFR, SRC, ESR1 and MAPK1 were recognized as the main potential targets involved in T2DM [40, 41]. Moreover, insulin resistance, hepatitis C, pancreatic cancer, TNF signaling pathway, insulin signaling pathway, and PI3K-AKT signaling pathway were the mechanisms by which apple pollen exerted its therapeutic effect on T2DM [38].

The above results reduce the prediction range and increase the accuracy of the prediction results, also providing important information for performing further pharmacological investigations on apple pollen.

## Figures and Tables

**Table 1:** Identification of chemical compositions in Qinguan pollen by HPLC-QTOF-MS/MS.

No.	*t*_*R*_/min	m/z	MS	Formula	MS_2_	Component
1	4.24	331.0815	330.0774 (M-H)^−^	C_17_H_15_O_7_	285, 251, 199, 145	Malvidin
2	4.44	182.0784	181.0744 (M-H)^−^	C_6_H_14_O_6_	182, 168, 146, 131	D-(-)-Mannitol
3	5.05	200.0619	199.033 (M-H)^−^	C_8_H_12_N_2_O_2_S	199, 187, 179, 146, 135	N,N-Dimethyl-n′-phenylsulfamide
4	6.90	196.0581	195.0508 (M-H)^−^	C_6_H_12_O_7_	195, 179, 146, 129	Gluconic acid
5	7.04	134.0214	133.0141 (M-H)^−^	C_4_H_6_O_5_	133, 127, 71, 62	Malic acid
6	7.56	226.1317	225.0160 (M-H)^−^	C_11_H_18_N_2_O	225, 209, 197, 181, 160	Amobarbital
7	18.79	280.0456	279.0383 (M-H)^−^	C_18_H_32_O_2_	279, 252, 233, 201, 180	Linoleic acid
8	19.55	162.0528	161.482 (M-H)^−^	C_6_H_10_O_5_	161, 146, 130, 96, 79	3-Hydroxy-3-methylglutaric acid
9	21.57	165.065	164.0743 (M-H)^−^	C_6_HN_5_O	164, 146, 129, 116, 96	7-Methylguanine
10	22.15	191.0582	189.8607 (M-H)^−^	C_10_H_9_NO_3_	189, 174, 164, 160, 129	5-Hydroxyindoleacetic acid
11	25.42	204.0898	203.0854 (M-H)^−^	C_11_H_12_N_2_O_2_	203, 197, 146, 129, 113, 100, 92	L-Tryptophan
12	29.31	326.1001	325.0924 (M-H)^−^	C_15_H_18_O_8_	325, 292, 258, 145, 117	Coumarinic acid-beta-D-glucoside
13	30.83	354.0945	353.0872 (M-H)^−^	C_16_H_18_O_9_	353, 322, 209, 191, 173	Chlorogenic acid
14	35.30	304.2013	303.1079 (M+H)^−^	C_20_H_32_O_2_	305, 213, 191, 173, 169	Arachidonic acid
15	40.95	164.0473	163.0395 (M-H)	C_9_H_8_O_3_	163154, 145, 119, 104, 93	m-Coumaric acid
16	42.67	166.0627	165.0554 (M-H)^−^	C_9_H_10_O_3_	165, 146, 129, 117, 101	DL-3-Phenyllactic acid
17	43.72	640.1638	639.1565 (M-H)^−^	C_28_H_32_O_17_	639, 514, 503, 459, 399314	Isorhamnetin-3-glucoside-4′-glucoside
18	53.60	436.1362	435.1289 (M-H)^−^	C_21_H_24_O_10_	435, 379, 273, 229, 179	Phloridzin
19	53.70	478.1105	477.1032 (M-H)^−^	C_22_H_22_O_12_	477, 435, 395, 357, 299	Isorhamnetin-3-O-glucosie
20	55.05	508.121	507.1137 (M-H)^−^	C_23_H_24_O_13_	507, 387, 359, 329, 258	Syringetin-3-O-galactoside
21	68.70	316.057	315.0505 (M-H)^−^	C_16_H_12_O_7_	255, 227, 193, 165, 136	Rhamnetin

**Table 2:** Identification of chemical compositions in Gala pollen by HPLC-QTOF-MS/MS.

No.	*t*_*R*_/min	m/z	MS	Formula	MS_2_	Component
1	4.21	132.0536	131.0463 (M-H)^−^	C_5_H_8_O_4_	131, 112	Glutaric acid
2	4.72	182.0786	181.0713 (M-H)^−^	C_6_H_14_O_6_	181, 146, 129, 96	Dulcitol
3	6.91	196.0947	195.0874 (M-H)^−^	C_6_H_12_O_7_	195, 174, 146, 133, 129	Gluconic acid
4	19.50	162.0527	161.0455 (M-H)^−^	C_6_H_10_O_5_	161, 146, 129, 93	3-Hydroxy-3-methylglutaric acid
5	22.05	287.0550	286.0478 (M-H)^−^	C_15_H_10_O_6_	287.0550, 153.0212	Kaempferol
6	30.83	354.0945	353.0872 (M-H)^−^	C_16_H_18_O_9_	353, 322, 209, 191, 173	Chlorogenic acid
7	44.81	640.1618	639.1654 (M-H)^−^	C_28_H_32_O_17_	639, 514, 362, 197	Isorhamnetin-3,4′-diglucoside
8	46.11	624.1687	623.1700 (M-H)^−^	C_28_H_32_O_16_	623, 498, 362, 248, 197	Isorhamnetin-3-galactoside-6″-rhamnoside
9	50.21	434.08491	433.0767 (M-H)^−^	C_20_H_18_O_11_	433, 301, 300, 271, 227	Quercitrin
10	55.01	508.1211	507.1138 (M-H)^−^	C_23_H_24_O_13_	507, 362, 329, 197	Syringetin-3-O-galactoside
11	58.01	418.09	417.0820 (M-H)^−^	C_20_H_18_O_10_	417, 357, 284, 255, 227	Kaempferol-3-O-alpha-L-arabinoside
12	69.52	316.0575	315.0502 (M-H)^−^	C_16_H_12_O_7_	255, 227, 193, 165, 136	Rhamnetin

**Table 3:** Absorption parameters of the components.

No.	Compounds	Pharmacokinetics	Druglikeness (yes ≥ 2)	Results
		GI absorption	Lipinski/Veber/Ghose/Egan/Muegge	
1	Malvidin	High	√	√
2	D-(-)-Mannitol	Low	√	×
3	N,N-Dimethyl-n′-phenylsulfamide	High	√	√
4	Gluconic acid	Low	√	×
5	Malic acid	High	√	√
6	Amobarbital	High	√	√
7	Linoleic acid	High	√	√
8	3-Hydroxy-3-methylglutaric acid	High	√	√
9	7-Methylguanine	High	√	√
10	5-Hydroxyindoleacetic acid	High	√	√
11	L-Tryptophan	High	√	√
12	Coumarinic acid-beta-D-glucoside	Low	×	×
13	Chlorogenic acid	Low	×	×
14	Arachidonic acid	High	√	√
15	DL-3-Phenyllactic acid	High	√	√
16	Isorhamnetin-3-glucoside-4′-glucoside	—	—	—
17	Phloridzin	Low	×	×
18	Isorhamnetin-3-O-glucoside	High	√	√
19	Syringetin-3-O-galactoside	Low	×	×
20	Rhamnetin	High	√	√
21	Glutaric acid	High	√	√
22	Dulcitol	Low	√	×
23	m-Coumaric acid	High	√	√
24	Isorhamnetin-3,4′-diglucoside	—	—	—
25	Isorhamnetin-3-galactoside-6′-rhamnoside	—	—	—
26	Quercitrin	High	√	√
27	Kaempferol-3-O-alpha-L-arabinoside	High	√	√
28	Kaempferol	High	√	√

## Data Availability

The data used to support the findings of this study are included within the article.
